# The first-trimester serum high-temperature requirement protease A4 and uterine artery Doppler for the prediction of preeclampsia

**DOI:** 10.1038/s41598-023-35243-z

**Published:** 2023-05-22

**Authors:** Patcharaporn Siricharoenthai, Vorapong Phupong

**Affiliations:** Placental Related Diseases Research Unit, Department of Obstetrics and Gynecology, Faculty of Medicine, Chulalongkorn University, Rama IV Road, Pathumwan, Bangkok, 10330 Thailand

**Keywords:** Biomarkers, Diseases

## Abstract

The objective of this study was to investigate the predictive value of serum high-temperature requirement protease A4 (HtrA4) and the first-trimester uterine artery in predicting preeclampsia in singleton pregnancy. Pregnant women at gestational age 11–13^+6^ weeks, who visited the antenatal clinic at King Chulalongkorn Memorial Hospital, Department of Obstetrics and Gynecology, Faculty of Medicine, Chulalongkorn University during April 2020–July 2021 were included. Serum HtrA4 levels and transabdominal uterine artery Doppler ultrasound were performed to evaluate this combination for calculating the predictive value of preeclampsia. While 371 singleton pregnant women enrolled in this study, 366 completed it. Thirty-four (9.3%) women had preeclampsia. Mean serum HtrA4 levels were higher in the preeclampsia group than in the control group (9.4 ± 3.9 vs 4.6 ± 2.2 ng/ml, p < 0.001). The mean uterine artery pulsatility index (UtA-PI) was higher in the group with early onset preeclampsia than in the control group (2.3 ± 0.5 vs 1.7 ± 0.5, p = 0.002). The sensitivity, specificity, positive predictive value (PPV), and negative predictive value (NPV) were 76.5%, 90.7%, 45.6%, and 97.4%, respectively, when using serum HtrA4 levels above 1.8 multiples of the median for the gestational age as a cut-off value for predicting preeclampsia. A combination of serum HtrA4 levels and UtA-PI > 95th percentile yielded sensitivity, specificity, PPV, and NPV of 79.4%, 86.1%, 37% and 97.6%, respectively, for the prediction of preeclampsia. A combination of serum HtrA4 levels and uterine artery Doppler in the first trimester had good sensitivity for predicting preeclampsia.

## Introduction

Preeclampsia is one of the three leading causes of maternal death worldwide. It also increases maternal and fetal morbidities^1,2^. Preeclampsia is a hypertensive disorder occurring during pregnancy that complicates with proteinuria and multiple organ involvement. Moreover, those who develop preeclampsia are at an increased risk of developing coronary heart disease and cerebrovascular disease later in their lifetime^3^. The global incidence is 2–8%, depending on race, environment and criteria for diagnosis^4,5^. In King Chulalongkorn Memorial Hospital, the five-year incidence was 4.99%^6^.

To date, many studies have investigated the pathogenesis of preeclampsia, but the definite cause has not been determined. The most accepted cause is an abnormal invasion of trophoblastic cells to the uterine spiral vessels. The imbalance between angiogenetic and immunologic factors also plays an important role in the development of preeclampsia^7,8^.

Early diagnosis can improve maternal and fetal outcomes along with reducing the use of medical facilities. Therefore, early identification of those who are at risk of developing preeclampsia is very important. Pregnant women who screened positive would benefit from the use of aspirin and close surveillance^5,9^. According to the American College of Obstetricians and Gynecologists (2019), maternal history can be used as a screening method with a yield of 30% sensitivity^9^. Recent studies showed that combined methods yielded higher sensitivity and specificity than maternal history alone^10–12^.

High-temperature requirement factor A4 (HtrA4) is the placenta-specific serine proteases which were detected in the maternal circulation of patients diagnosed with preeclampsia. The exact role of HtrA4 is still understudied. The serine protease family includes HtrA1, HtrA2, HtrA3, and HtrA4. All HtrAs play roles in the regulation of cellular processes such as cell proliferation, stress response, and programmed cell death. HtrA4, the latest member of the family, expresses mostly at the placenta and secretes to the maternal circulation. Its level continues to rise until 24–25 weeks of gestation and remains stable throughout pregnancy. In vitro and in vivo of previous studies showed a correlation between HtrA4 level and early-onset preeclampsia^13,14^. Thus, the up-regulation of HtrA4 expression may be involved in the etiology of pre-eclampsia, particularly in early-onset disease^13,14^.

Currently, studies on the use of uterine artery Doppler are increasing. The abnormally developed spiral arteries result in high impedance of uterine arteries. First-trimester uterine artery pulsatility index (UtA-PI) and presence of notch are good markers for identification of those with a high impedance of uterine arteries, which may be used as a screening test for preeclampsia^4^.

There has been no study on this combination being used as a screening test for preeclampsia, and it might increase sensitivity and specificity rather than a single tool. Thus, the aim of this study was to investigate the role of first-trimester serum HtrA4 levels combined with UtA-PI in predicting preeclampsia in singleton pregnancy.

## Materials and methods

This prospective observational study was performed at the Department of Obstetrics and Gynecology, Faculty of Medicine, Chulalongkorn University, King Chulalongkorn Memorial Hospital, Bangkok, Thailand, between April 2020 and July 2021. The study was approved by the Institutional Review Board of the Faculty of Medicine, Chulalongkorn University. This study has been performed in accordance with the Declaration of Helsinki. All women received information about the study protocol and provided written informed consent prior to any procedures.

The inclusion criteria were singleton pregnant women aged 20–45 years old and gestational age of 11–13^+6^ weeks. The exclusion criteria were pregnant women who carried an abnormal fetus (structural or chromosomal) and those who took aspirin prior to enrollment in the study. If there is an indication for using prophylactic aspirin, aspirin has been started after the sampling and sonographic evaluation.

Sample size calculation was based upon the expected sensitivity of the test at 80%, with 20% allowable error and α error at 0.05. Prevalence was based on the preeclampsia incidence at King Chulalongkorn Memorial Hospital in the past five years, which was 4.99%. We need 16 preeclamptic cases to test this hypothesis. A minimum of 368 women were required in this study, for adjustments of 4.99% incidence of preeclampsia at our institute and a loss to follow-up rate of 15%,

Preeclampsia is defined by a new onset hypertension (an elevated systolic blood pressure (SBP) of at least 140 mmHg or diastolic blood pressure (DBP) of at least 90 mmHg), measured on two occasions at least 6 h apart, combined with proteinuria (at least 1 + on urine dipstick test or 300 mg/24 h or urine protein/creatinine ratio index ≥ 0.3) after 20 weeks of gestation. In the absence of proteinuria, the presence of headache, visual disturbance, epigastric pain, pulmonary edema, increased creatinine level, thrombocytopenia, and transaminitis was also considered preeclampsia^8^.

The primary aim was to investigate the predictive value of first-trimester serum HtrA4 combined with UtA-PI in predicting preeclampsia. The secondary aim was to identify the predictive value of this combination to detect adverse pregnancy outcomes, such as preterm delivery, gestational diabetes, and fetal growth restriction.

Maternal demographic data, UtA-PI, the presence of uterine artery notching, and maternal and neonatal outcomes were recorded. Blood pressure was measured by validated automated devices (Microlife AG, 9443 Widnau, Switzerland) after resting in a sitting position for 5 min. The SBP, DBP, and mean arterial pressure (MAP) were recorded.

Transabdominal ultrasound was performed by a single investigator to assess uterine artery Doppler using ultrasonographic machines with a convex probe AB 2–7 MHz (GE Voluson E10, GE Medical Systems, Zipf, Austria). After the mid-sagittal plane of the uterus and cervix was obtained, the probe was then tilted laterally to demonstrate uterine artery along both sides. A 2 mm gate pulse-wave Doppler was positioned on the uterine artery at the level of the internal os of the cervix, with an insonation angle of less than 30°. Three waveforms with a peak systolic velocity > 60 cm/s were obtained. Each side was measured three times, and the mean of the UtA-PI was calculated and recorded. The presence or absence of the uterine artery notch was recorded for each side^15^.

Blood for serum HtrA4 was drawn via venipuncture and collected in non-heparinized tubes before centrifugation at 2,500 rpm for 10 min and stored at -80 °C until assayed. Serum samples were collected at 8.00–10.00 a.m. All the samples were analyzed in the same period after finishing patient enrollment by ELISA Kit test for HtrA4 (Cloud-Clone Corp, China), which is a sandwich enzyme immunoassay. It was used to measure maternal serum HtrA4 levels, with the ranges of intra- and inter-assay < 10%.

Statistical analysis was performed using SPSS software version 22.0 (IBM, New York, USA). Data were presented in mean, standard deviation (SD), median, interquartile range (IQR), sensitivity, specificity, positive predictive value (PPV), and negative predictive value (NPV) with a 95% confidence interval (CI). The cut-off value of serum HtrA4 level was calculated by using the receiver operating characteristic (ROC) curve. The chi-square test was used for comparing categorical data, while the independent t-test and Mann Whitney U test were used for comparing continuous data. A p value < 0.05 was considered statistically significant.

## Results

**Figure 1:**
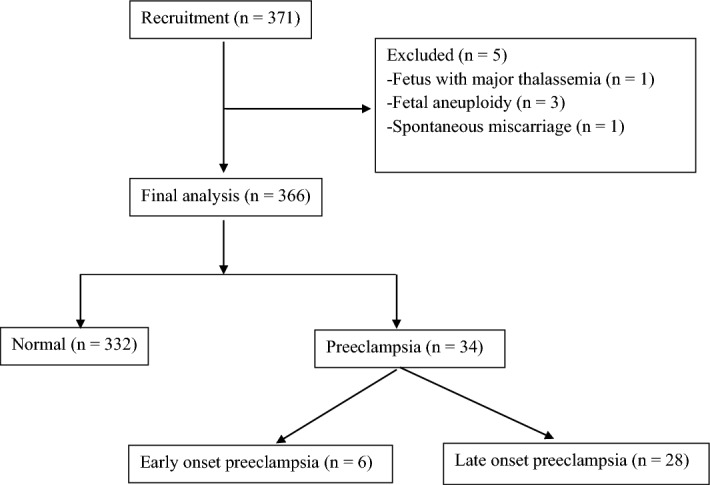
Study flow chart.

A total of 371 pregnant women were enrolled in the study. Five women were excluded (one case with major thalassemia, three cases with aneuploidy, and one case with spontaneous miscarriage). The data from a total of 366 women were analyzed (Fig. 1). Thirty-four cases were diagnosed with preeclampsia (9.3%) and 6 (1.6%) had early-onset preeclampsia. Fourteen patients (3.8%) were diagnosed with severe preeclampsia. There was no difference between the gestational age at enrollment, parity, size of fetus estimated by crown-rump length (CRL), maternal weight gain, and pre-pregnancy diabetes mellitus of pregnant women with preeclampsia and normal pregnant women. Maternal age, pre-pregnancy body mass index (BMI), chronic hypertension, and MAP were significantly higher in the preeclampsia group than in the control group (Table 1).

**Table 1:** Demographic data, maternal and neonatal outcomes of women with preeclampsia compared with women without preeclampsia.

	Control (n = 332)	Preeclampsia (n = 34)	p value
Maternal age (years)	32.9 ± 4.5	35.1 ± 3.9	< 0.001
Advanced maternal age (≥ 35 years)	125 (37.7)	22 (64.7)	0.002
Primigravida	168 (50.6)	17 (50)	0.946
Nulliparous	202 (60.8)	20 (58.8)	0.818
Prepregnancy BMI (kg/m^2^)	22.4 ± 3.9	25.6 ± 6.4	< 0.001
Overt DM	4 (1.2)	2 (5.9)	0.099
Chronic hypertension	3 (0.9)	8 (23.5)	< 0.001
Obesity (BMI ≥ 30 kg/m^2^)	18 (5.4)	8 (16)	0.006
Total weight gain (kg)	13.3 ± 4.6	13.9 ± 5.7	0.44
GA at measurement (weeks)	12.3 ± 0.7	12.3 ± 0.6	0.924
CRL (mm)	65.5 ± 9.4	66.6 ± 7.5	0.483
Mean arterial pressure (mmHg)	84.4 ± 9.4	91.9 ± 10.7	< 0.001
Gestational diabetes	28 (8.4)	3 (8.8)	1.000
Fetal growth restriction	1 (0.3)	7 (20.6)	< 0.001
Preterm delivery	29 (8.7)	11 (32.4)	< 0.001
GA at delivery (weeks)	37.9 ± 1.9	36.6 ± 2.5	0.003
Delivery at GA < 37 weeks	29 (8.7)	11 (32.4)	< 0.001
Delivery at GA < 34 weeks	8 (2.4)	3 (8.8)	0.072
Mode of delivery			0.563
Vaginal delivery	114 (34.3)	10 (29.4)	
Cesarean section	218 (65.7)	24 (70.6)	
Birth weight (grams)	3055.1 ± 492.5	2784.9 ± 729.3	0.004
Low birth weight (< 2500 g)	22 (6.6)	7 (20.6)	0.004
Apgar score			
1 min < 7	6 (1.8)	3 (8.8)	0.041
5 min < 7	2 (0.6)	1 (2.9)	0.254
Neonatal respiratory distress syndrome	4 (1.2)	4 (11.8)	0.003
Perinatal death	2 (0.6)	1 (2.9)	0.254
Intraventricular hemorrhage	2 (0.6)	0	1.000
Sepsis	10 (3.0)	1 (2.9)	1.000
Median length of hospital stays	3 (3, 4)	4 (3, 5.3)	0.022

Data are presented in mean ± standard deviation or n (%).

*BMI* body mass index, *DM* diabetes mellitus, *GA* gestational age, *CRL* crown-rump length.

Pregnant women with preeclampsia had lesser gestational age at delivery than the controls, while the Apgar score and perinatal death were not different. The rate of fetal growth restriction and respiratory distress syndrome (RDS) in the preeclampsia group was significantly higher (Table 1).

**Figure 2:**
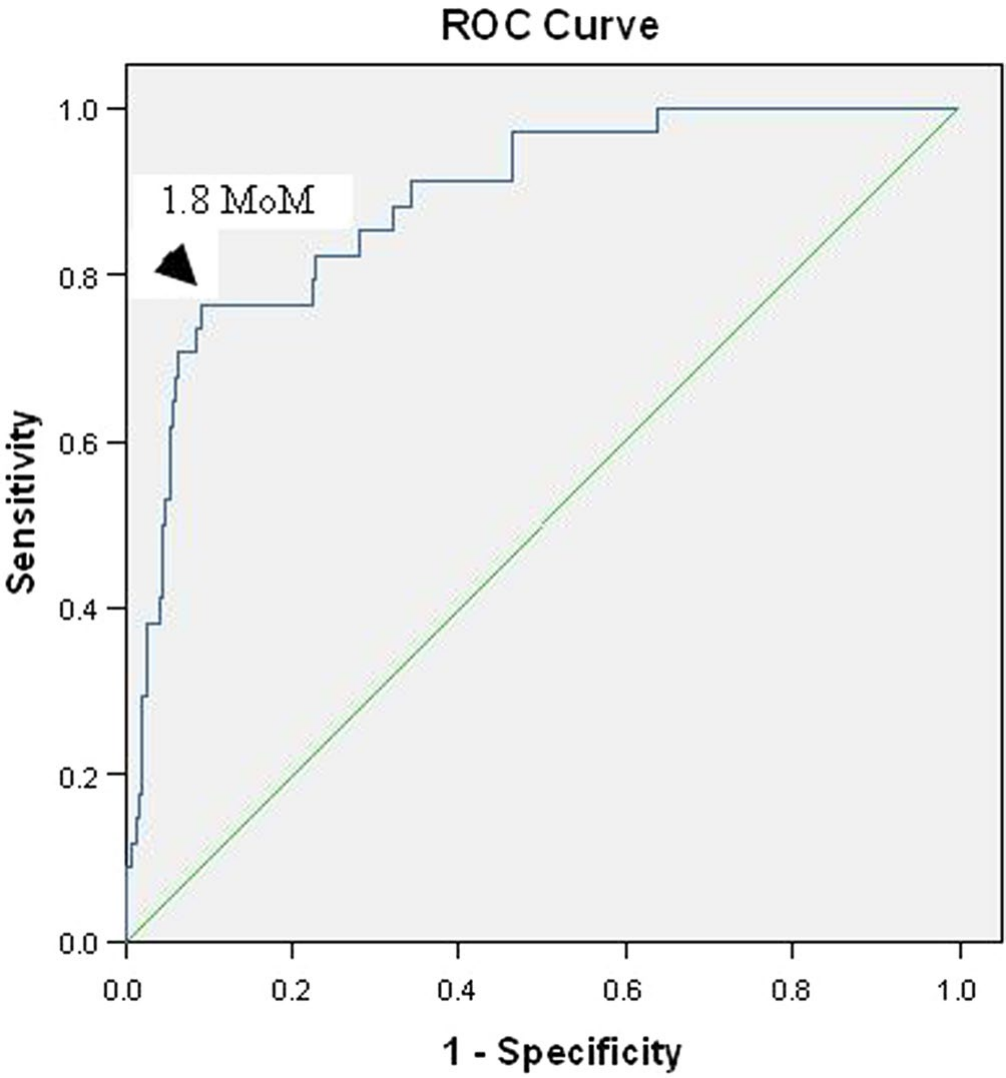
Receiver-operating characteristic curve for the relationship between the serum high temperature requirement factor A4 (Htr A4) levels and the diagnosis of preeclampsia [area under curve (AUC) 0.886, p < 0.001, 95% confidence interval 0.830–0.942].

The mean serum HtrA4 levels were higher in the preeclampsia group than the control group (9.4 ± 3.9 vs 4.6 ± 2.2 ng/ml, p < 0.001). Higher levels were observed in the early-onset preeclampsia group at 11.3 ± 3.8 ng/ml (p < 0.001) (Table 2). After running the ROC curve with AUC 0.886 (Fig. 2), the cut-off value of the HtrA4 level was 1.8 multiples of median (MoM). This cut-off value yielded sensitivity, specificity, PPV, and NPV of 76.5%, 90.7%, 45.6%, and 97.4%, respectively, for predicting preeclampsia.

**Table 2:** Serum HtrA4 levels and UtA-PI in overall, early-onset, and late-onset preeclampsia compared with control healthy women.

	Control (n = 332)	Preeclampsia (n = 34)	Early-onset preeclampsia (n = 6)	Late-onset preeclampsia (n = 28)	p value
HtrA4 (ng/ml)	4.6 ± 2.2	9.4 ± 3.9			< 0.001
			11.3 ± 3.8		< 0.001
				9.0 ± 3.8	< 0.001
Median of HtrA4 (ng/ml)	4.1 (3.0, 5.7)	9.0 (7.1, 10.3)			< 0.001
			10.3 (8.7, 12.7)		< 0.001
				8.7 (5.7, 10)	< 0.001
UtA-PI	1.7 ± 0.5	1.8 ± 0.6			0.435
			2.3 ± 0.5		0.002
				1.6 ± 0.6	0.607
Any notching	210 (63.3)	28 (82.4)			0.026
			6 (100)		0.091
				22 (78.6)	0.103
Bilateral notching	150 (45.2)	25 (73.5)			0.002
			6 (100)		0.009
				19 (67.9)	0.02

Data are presented in median (interquartile range), mean ± standard deviation or n (%).

*HtrA4* high-temperature requirement factor A4, *UtA-PI* uterine artery pulsatility index.

The sensitivity, specificity, PPV, and NPV were 14.7%, 95.2%, 23.8%, and 91.6%, respectively, when using the mean UtA-PI above the 95th percentile, according to the gestational age at the time of measurement as a cut-off value for predicting preeclampsia (Table 3). A significant difference in mean UtA-PI was observed in the early-onset preeclampsia group (2.3 ± 0.5 vs 1.7 ± 0.5, p = 0.002).

**Table 3:** Predictive value of serum HtrA4 levels and uterine artery Doppler for preeclampsia.

	Sensitivity (%)	Specificity (%)	PPV (%)	NPV (%)	Positive LR	Negative LR
Overall preeclampsia						
HtrA4 levels > 1.8 MoM	76.5 (58.8–89.3)	90.7 (87.0–93.6)	45.6 (36.4–55.2)	97.4 (95.4–98.6)	8.2 (5.6–12.0)	0.3 (0.1–0.5)
UtA-PI > 95th percentile	14.7 (4.9–31.1)	95.2 (92.3–97.2)	23.8 (10.9–44.5)	91.6 (90.4–92.6)	3.1 (1.2–7.8)	0.9 (0.8–1.0)
Abnormal HtrA4 levels and/or UtA-PI	79.4 (62.1–91.3)	86.1 (81.9–89.7)	37.0 (29.9–44.7)	97.6 (95.5–98.8)	5.7 (4.2–7.9)	0.2 (0.1–0.5)
Early-onset preeclampsia						
HtrA4 levels > 1.8 MoM	100 (54.1–100)	90.7 (87.0–93.6)	16.2 (12.2–21.3)	100	10.7 (7.7–15)	0
UtA-PI > 95th percentile	33.3 (4.3–77.7)	95.2 (92.3–97.2)	11.1 (3.5–29.9)	98.8 (97.8–99.3)	6.9 (2.0–23.6)	0.7 (0.4–1.2)
Abnormal HtrA4 levels and/or UtA-PI	100 (54.1–100)	86.1 (82–89.7)	11.5 (9.1–14.6)	100	7.2 (5.5–9.4)	0
Late-onset preeclampsia						
HtrA4 levels > 1.8 MoM	71.4 (51.3–86.8)	90.7 (87.0–93.6)	39.2 (30.0–49.3)	97.4 (95.4–98.5)	7.7 (5.1–11.5)	0.3 (0.2–0.6)
UtA-PI > 95th percentile	10.7 (2.3–28.2)	95.2 (92.3–97.2)	15.8 (5.5–37.7)	92.7 (91.7–93.5)	2.2 (0.7–7.2)	0.9 (0.8–1.1)
Abnormal HtrA4 levels and/or UtA-PI	75 (55.1–89.3)	86.1 (82–89.7)	31.3 (24.5–39.2)	97.6 (95.6–98.3)	5.4 (3.8–7.6)	0.8 (0.3–0.6)

*HtrA4* high-temperature requirement factor A4, *UtA-PI* uterine artery pulsatility index, *PPV* positive predictive value, *NPV* negative predictive value, *LR* likelihood ratio.

After combining serum HtrA4 levels above 1.8 MoM with UtA-PI above the 95th percentile, predictive value increased more than using these two tests separately. Sensitivity, specificity, PPV, and NPV were 79.4%, 86.1%, 37%, and 97.6%, respectively. Moreover, in early-onset preeclampsia, the sensitivity, specificity, PPV, and NPV were 100%, 86.1%, 11.5%, and 100%, respectively (Table 3).

Using this combination, the relative risk for preterm delivery, gestational diabetes mellitus, and fetal growth restriction were 1.2, 1.0, and 1.1, respectively (Table 4). The relative risk was not significant for preterm delivery, gestational diabetes mellitus, and fetal growth restriction after using the combination of serum HtrA4 levels and uterine artery Doppler in the first trimester.

**Table 4:** Serum HtrA4 levels and uterine artery Doppler for other pregnancy complications.

	Relative risk	95% confidence interval
Preterm delivery	1.2	1.1–1.4
Gestational diabetes	1.0	0.9–1.1
Fetal growth restriction	1.1	1.0–1.2

*HtrA4* high-temperature requirement factor A4.

## Discussion

This study found that the combination of serum HtrA4 levels and uterine artery Doppler in the first trimester was effective for predicting preeclampsia.

The serum HtrA4 level was higher in the preeclampsia group than in the control group (9.4 vs 4.6 ng/ml). HtrA4 was overexpressed, particularly in syncytiotrophoblast in preeclamptic placentas, and maternal serum HtrA4 levels were elevated in preeclampsia gestations^14^. The high level of placental-derived HtrA4 is a possible causal factor of endothelial dysfunction. HtrA4 profoundly altered human umbilical vein endothelial cells expression of several factors essential for normal endothelial cell function and inflammation responses^16^. An early study by Inagaki et al. (2012), that tested for serum HtrA4 level in preeclampsia women, showed a cut-off value of 139.5 ng/ml, which allowed for high sensitivity (94.7%) and high specificity (78.0%)^14^. However, the serum HtrA4 level in this study had lower levels than that of the study by Inagaki et al. This difference may be due to the difference in gestational age at blood collection and study population.

This study found that serum HtrA4 levels were elevated before the onset of preeclampsia, making it one of the possible biomarkers that can predict preeclampsia. Sensitivity and specificity of serum HtrA4 levels in the first trimester to predict preeclampsia in this study were 76.5% and 90.7%, respectively. The previous study found that the mean platelet volume (MPV), the neutrophil to lymphocyte ratio (NLR), and the platelet to lymphocyte ratio (PLR) values in the first trimester were significantly higher in patients who developed preeclampsia in later pregnancy weeks. The optimal cut-off value for MPV was 10.65 fL, with a sensitivity of 63.7% and a specificity of 65.0%. The best predictor for preeclampsia was NLR at an optimal cut-off value of 4.12, with a sensitivity of 82.1% and specificity of 62.0%. At a cut-off value of 131.8, PLR predicted preeclampsia with a sensitivity rate of 65.0% and a specificity rate of 60.2%^17^. One study found that serum aquaporin-9 concentrations increased significantly in early-onset preeclampsia compared to healthy normotensive pregnant patients, suggesting that aquaporin-9 might be a crucial biomarker of the inflammatory process in early-onset preeclampsia^18^.

The mean pulsatility index of the uterine artery Doppler in the present study was significantly higher in pregnant women with early-onset preeclampsia than that in the women in the control group. In this study, the method of using uterine artery Doppler for predicting preeclampsia and early-onset preeclampsia had high specificity (91.6% and 98.8%, respectively) but low sensitivity (14.7% and 33.3%, respectively). A recent meta-analysis also showed similar results; uterine artery Doppler had low sensitivity (26.4% and 47.8%, respectively) but high specificity (93.4% and 92.1%, respectively) in all preeclampsia and early-onset preeclampsia, respectively^19^.

With a combination of serum HtrA4 levels above 1.8 MoM and UtA-PI above 95th percentile, the sensitivity increased to 79.4% and specificity to 86.1%. Moreover, in early-onset preeclampsia, the sensitivity was 100% and specificity 86.1%. This first-trimester combination tests allowed us to screen with higher sensitivity compared with using a single test alone, especially in early-onset preeclampsia. This finding was consistent with previous studies that had high sensitivity to predict overall preeclampsia^10,11^ and early-onset preeclampsia^10^. Some studies demonstrated that the first-trimester combination of UtA-PI with maternal serum markers could only predict early-onset preeclampsia^20,21^, but it had poor sensitivity in predicting preeclampsia^20,21^.

The study population consisted of low- and high-risk pregnancies. The results for the predictive values of HtrA4 could have been stronger if pregnancies with chronic hypertension and overt diabetes were excluded. However, this is the real-world situation where there are low- and high-risk pregnancies. Thus, this combination test of serum HtrA4 levels and UtA-PI can be applied for use in all low- and high-risk pregnancies.

The strength of this study is that it is the first prospective study using the first-trimester serum HtrA4 levels combined with the UtA-PI to predict preeclampsia. The results from this early combined screening may allow patients at high risk to start aspirin prophylaxis to prevent preeclampsia. This may be more effective if started between 12 and 28 weeks of gestation^22^. The limitation was that this study had a few cases of early-onset preeclampsia. Further studies with a larger sample size of early-onset preeclampsia should be conducted to confirm its effectiveness in predicting early-onset preeclampsia.

## Conclusion

This study demonstrated that the combination of serum HtrA4 levels and uterine artery Doppler in the first-trimester was effective for predicting preeclampsia.

## Data Availability

The datasets generated during and/or analyzed during the current study are not publicly available due to the permission of the Internal Review Board but are available from the corresponding author on reasonable request.
